# Biofilm Matrix Regulation by *Candida albicans* Zap1

**DOI:** 10.1371/journal.pbio.1000133

**Published:** 2009-06-16

**Authors:** Clarissa J. Nobile, Jeniel E. Nett, Aaron D. Hernday, Oliver R. Homann, Jean-Sebastien Deneault, Andre Nantel, David R. Andes, Alexander D. Johnson, Aaron P. Mitchell

**Affiliations:** 1 Department of Microbiology, Columbia University, New York, New York, United States of America; 2 Department of Microbiology and Immunology, University of California San Francisco, San Francisco, California, United States of America; 3 Department of Medicine, University of Wisconsin, Madison, Wisconsin, United States of America; 4 Biotechnology Research Institute, National Research Council of Canada, Montreal, Quebec, Canada; 5 Department of Biological Sciences, Carnegie Mellon University, Pittsburgh, Pennsylvania, United States of America; University of Aberdeen, United Kingdom

## Abstract

A biofilm is a surface-associated population of microorganisms embedded in a matrix of extracellular polymeric substances. Biofilms are a major natural growth form of microorganisms and the cause of pervasive device-associated infection. This report focuses on the biofilm matrix of *Candida albicans*, the major fungal pathogen of humans. We report here that the *C. albicans* zinc-response transcription factor Zap1 is a negative regulator of a major matrix component, soluble β-1,3 glucan, in both in vitro and in vivo biofilm models. To understand the mechanistic relationship between Zap1 and matrix, we identified Zap1 target genes through expression profiling and full genome chromatin immunoprecipitation. On the basis of these results, we designed additional experiments showing that two glucoamylases, Gca1 and Gca2, have positive roles in matrix production and may function through hydrolysis of insoluble β-1,3 glucan chains. We also show that a group of alcohol dehydrogenases Adh5, Csh1, and Ifd6 have roles in matrix production: Adh5 acts positively, and Csh1 and Ifd6, negatively. We propose that these alcohol dehydrogenases generate quorum-sensing aryl and acyl alcohols that in turn govern multiple events in biofilm maturation. Our findings define a novel regulatory circuit and its mechanism of control of a process central to infection.

## Abstract 2

The zinc-responsive transcription factor Zap1 has a striking role in fungal biofilm formation and is reported to regulate matrix formation.

## Author Summary

A biofilm is a surface-associated population of microbes that is embedded in a cement of extracellular compounds. This cement is known as matrix. The two main functions of matrix are to protect cells from their surrounding environment, preventing drugs and other stresses from penetrating the biofilm, and to maintain the architectural stability of the biofilm, acting as a glue to hold the cells together. The presence of matrix is a contributing factor to the high degree of resistance to antimicrobial drugs observed in biofilms. Because biofilms have a major impact on human health, and because matrix is such a pivotal component of biofilms, it is important to understand how the production of matrix is regulated. We have begun to address this question in the major human fungal pathogen *Candida albicans.* We found that the zinc-responsive regulatory protein Zap1 controls the expression of several genes important for matrix formation in *C. albicans*. These target genes encode glucoamylases and alcohol dehydrogenases, enzymes that probably govern the synthesis of distinct matrix constituents. The findings here offer insight into the metabolic processes that contribute to biofilm formation and indicate that Zap1 functions broadly as a negative regulator of biofilm maturation.

## Introduction

A biofilm is a community of surface-associated microorganisms embedded in a matrix of extracellular polymeric substances. Biofilms are common microbial growth forms in nature and are a leading cause of human infection [1]. These infections are seeded from biofilms present on implanted medical devices, such as intravascular catheters [2]. Biofilm formation mechanisms are thus relevant to our understanding of both microbial ecology and infectious disease.

Biofilm matrix is broadly defined as an extracellular polymeric material that is maintained within a biofilm [3]–[6]. It derives from directed synthesis and secretion of matrix components as well as lysis of a fraction of biofilm cells [5]. In natural settings, matrix constituents may also come from the local environment, such as an infected host [5]. Biofilm matrix often consists predominantly of extracellular polysaccharides. For example, bacterial biofilm matrices can include cellulose, polysaccharide intercellular adhesin, and the polysaccharide polymers VPS, PEL, and PSL [6]. Other matrix components include proteins, fatty acids, and nucleic acids [6],[7]. In general, the matrix provides support and protection of the microbial community embedded within it.

Our focus is the biofilm matrix of *C. albicans*, the major fungal pathogen of humans. The *C. albicans* matrix is composed primarily of carbohydrate and includes protein, hexosamine, phosphorus, and uronic acid [8]. The primary carbohydrate is probably β-1,3 glucan: glucose is the major matrix sugar and biofilms are disrupted by in situ treatment with lyticase [8], an enzyme that specifically hydrolyzes β-1,3 glucan. Moreover, Nett et al. have shown that elevated β-1,3 glucan levels are characteristic of biofilm cells as compared to planktonic free-living *C. albicans* cells [9]. The increased β-1,3 glucan content of in vitro-grown biofilms is found in both cell walls and as a secreted form [9]. Finally, soluble β-1,3 glucan is produced by *C. albicans* biofilms grown in an in vivo catheter infection model, where it can be used in diagnosis of catheter-based infection [10].

Matrix production is closely tied to biofilm formation, yet little is known about its regulation or production mechanisms. We describe here a *C. albicans* transcription factor, Zap1/Csr1 (orf19.3794), that governs matrix production. This transcription factor is closely related to the *Saccharomyces cerevisiae* zinc-response regulator Zap1, and we show that expression of three zinc transporter genes depends upon *C. albicans* Zap1/Csr1. This observation supports a recent report [11] indicating that the *S. cerevisiae* and *C. albicans* Zap1 both regulate zinc-responsive gene expression. However, we also show that Zap1/Csr1 controls genes that influence overall matrix levels. Our results provide a foundation for a mechanistic understanding of matrix production and its regulation.

## Results

### Role of Zap1 in Biofilm Formation In Vitro

We have described screens of *C. albicans* transcription factor gene insertion mutants for defects in biofilm formation [12]. In the course of these screens, we found an insertion mutant that produced a biofilm with a slimy or glistening appearance. The insertion lay in the coding region for *ZAP1/CSR1* (orf19.3794). This phenotype was observed for several additional *zap1/zap1* insertion mutants as well as a newly created *zap1*Δ*/zap1*Δ deletion mutant. This unusual phenotype was complemented by introduction of a wild-type *ZAP1* construct into the *zap1*Δ*/zap1*Δ mutant, but not by the vector lacking the *ZAP1* insert. Therefore, loss of *ZAP1* function causes an unusual glistening appearance of in vitro-grown *C. albicans* biofilms.

**Figure 1:**
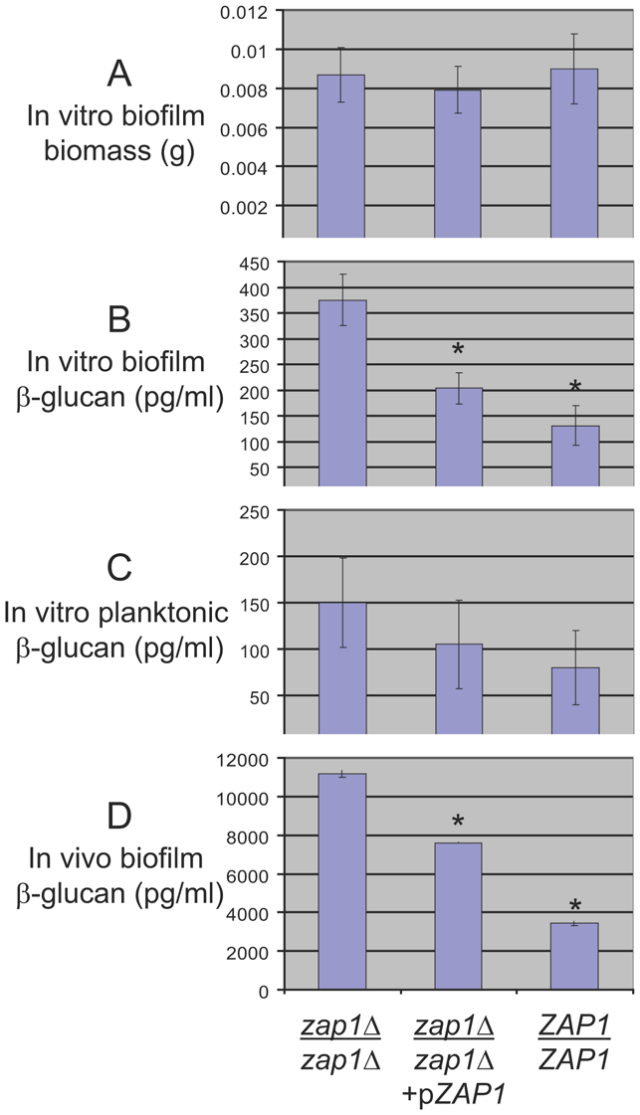
Analysis of biofilm and matrix production. The mutant strain CJN1201 (*zap1*Δ*/zap1*Δ), complemented strain CJN1193 (*zap1*Δ*/zap1*Δ+*pZAP1*), and reference wild-type strain DAY185 (*ZAP1/ZAP1*) were assayed for (A) in vitro-grown biofilm biomass, (B) in vitro-grown biofilm soluble β-1,3 glucan production, and (C) in vitro planktonic culture soluble β-1,3 glucan production. In addition, (D) soluble β-1,3 glucan production was assayed in a rat catheter biofilm infection model. The symbol “*” indicates that glucan measurements were significantly different (*p*<0.0005) from the *zap1*Δ*/zap1*Δ strain.

**Figure 2:**
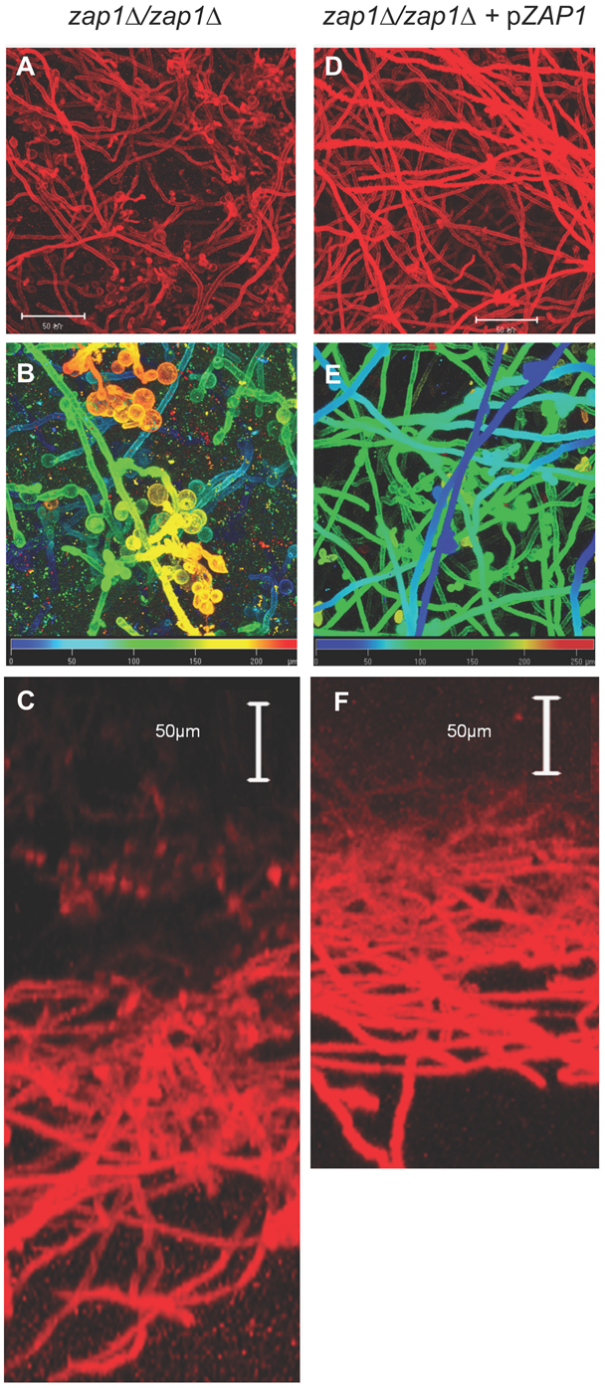
CSLM analysis of in vitro biofilm structure. In vitro-grown biofilms of the mutant strain CJN1201 (*zap1*Δ*/zap1*Δ, [A–C]) and complemented strain CJN1193 (*zap1*Δ*/zap1*Δ+*pZAP1*, [D–F]) were visualized by CSLM. (A,D) Depth views show the *x*-*y* plane. (B, E) Magnified depth views with pseudocolor scale. (C, F) Side views show the *y*-*z* plane.

We examined overall biofilm growth and ultrastructure to explore the nature of this altered biofilm appearance. We detected no difference in biofilm biomass of *zap1*Δ*/zap1*Δ mutant and the *zap1*Δ*/zap1*Δ+p*ZAP1* complemented strain or the reference wild-type strain (Figure 1A). Overall biofilm thickness was similar for the *zap1*Δ*/zap1*Δ mutant and the *zap1*Δ*/zap1*Δ+p*ZAP1* complemented strain as well (Figure 2C, 2F), as visualized by confocal scanning laser microscopy (CSLM). However, depth views revealed that the mutant hyphae often terminated in yeast-form cells (Figure 2A, 2B). Some of these cells appeared spherical and resembled chlamydospores. Complementation with *ZAP1* (Figure 2D, 2E) restored an appearance similar to wild-type biofilms in this system [12]. Therefore, Zap1 is required for normal hyphal morphogenesis in biofilms.

A glistening appearance can be associated with accumulation of extracellular polymers, as in the case of *Staphylococcus* biofilms [13]. To see whether matrix might hyperaccumulate in the *zap1*Δ*/zap1*Δ strain, we measured biofilm-associated soluble β-1,3 glucan. The *zap1*Δ*/zap1*Δ strain produced 1.5- to 2-fold greater soluble β-1,3 glucan in biofilms than the complemented and reference strains (Figure 1B). Planktonic cultures of the strains showed a similar trend but the differences were not statistically significant (Figure 1C). Therefore, in in vitro-grown biofilms, Zap1 is a negative regulator of extracellular soluble β-1,3 glucan, a major component of extracellular matrix.

### Role of Zap1 in Biofilm Formation In Vivo

**Figure 3:**
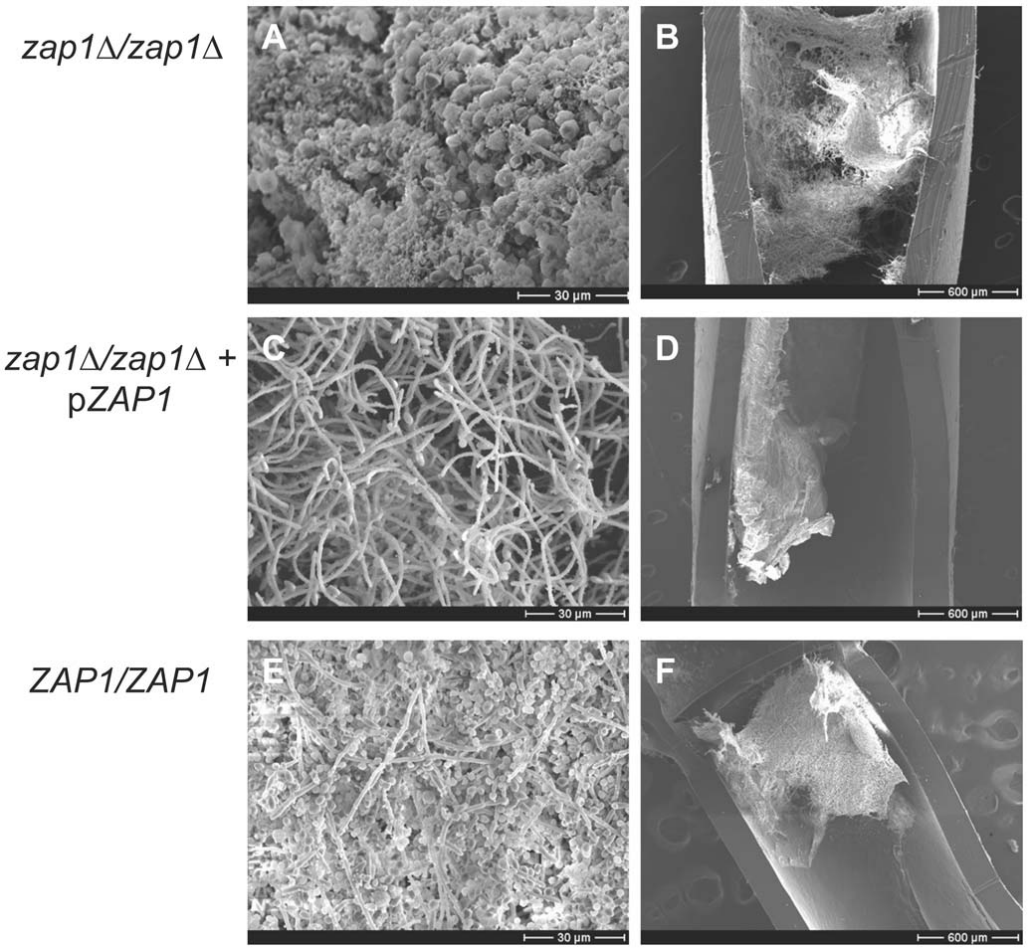
Scanning electron microscopy of in vivo biofilms. The mutant strain CJN1201 (*zap1*Δ*/zap1*Δ, [A,B]), complemented strain CJN1193 (*zap1*Δ*/zap1*Δ+*pZAP1*, [C,D]), and reference wild-type strain DAY185 (*ZAP1/ZAP1*, [E,F]) were inoculated into rat intravenous catheters, and resulting biofilms were visualized after 24 h of growth. Images show catheter luminal surfaces at (A,C,E) 1,000× and (B,D,F) 50× magnification.

In order to determine whether Zap1 may play a role in biofilm formation in vivo, we turned to a rat model for catheter-associated infection [14]. We observed that the *zap1*Δ*/zap1*Δ mutant, the *zap1*Δ*/zap1*Δ+p*ZAP1* complemented strain, and the wild-type reference strain all produced substantial biofilms in vivo (Figure 3B, 3D, 3F), as visualized with scanning electron microscopy (SEM). However, the *zap1*Δ*/zap1*Δ mutant biofilm had a striking abundance of extracellular material (Figure 3A) compared to the control strains (Figure 3C, 3E). Quantitative measurements of serum removed from the catheters indicated that the *zap1*Δ*/zap1*Δ mutant produced over 3-fold more soluble β-1,3 glucan than the wild-type strain (Figure 1D). Introduction of *ZAP1* into the mutant reduced soluble β-1,3 glucan production substantially (Figure 1D), as expected from the common phenomenon of partial complementation. These results indicate that Zap1 is a negative regulator of extracellular matrix production in an in vivo biofilm model.

### Identification of Zap1-Regulated Genes

In order to understand the connections between Zap1 and matrix production, we performed expression microarrays comparing the *zap1*Δ*/zap1*Δ mutant and complemented strain, both grown as biofilms. We found 232 genes that were significantly upregulated in the mutant, and 272 genes that were significantly downregulated genes in the mutant (Table 1; Dataset S4, worksheet 2). Several top target genes identified by the expression arrays were verified by northern or quantitative real-time PCR analysis (Dataset S5). The data indicate that *C. albicans* Zap1, like its *S. cerevisiae* ortholog, is a regulator of zinc homeostasis as the zinc transporter genes *ZRT1*, *ZRT2*, and *ZRT3* are downregulated in the *zap1*Δ*/zap1*Δ mutant. Indeed, we found that the *zap1*Δ*/zap1*Δ mutant is defective in growth on low-zinc medium (Dataset S5). That defect arises from reduced expression of zinc transporters, because increased expression of zinc transporter genes *ZRT1* or *ZRT2* improved growth of the *zap1*Δ*/zap1*Δ mutant on low-zinc medium (Dataset S5). These growth assays confirm findings reported recently by Kim et al. [11]. Several other gene classes are downregulated in the *zap1*Δ*/zap1*Δ mutant, including those related to adhesion, aldehyde metabolism, and hyphal development. The connection of adhesion and hyphal formation to biofilm formation is well established; the connection with aldehyde metabolism genes is discussed below. The classes of genes upregulated in the mutant include those related to alcohol dehydrogenase activity, carbohydrate transport, cell wall structure, ergosterol biosynthesis, and glucoamylase activity. The connection of several of these gene classes to biofilm formation is explored below. Finally, we note that the *zap1*Δ*/zap1*Δ strain has altered expression of several transcriptional regulatory genes, and these gene products may mediate indirect control of some genes by Zap1.

**Table 1:** Selected Zap1 regulated genes.

Systematic Name^a^	Gene Name^a^	log2 Expression Ratio^b^ (Complemented Strain/Mutant Strain)	Overexpressed^c^	ChIP-chip Target^d^	Zap1 Motif^e^	Description	GO Term or Other Descriptor^f^
orf19.3111	*PRA1*	5.73	In *zap1-*	Y	1 and 2	pH-Regulated antigen	Biological adhesion
orf19.3794	*CSR1 (ZAP1)*	4.87	—	Y	1 and 2	Zinc finger transcription factor	Zap1
orf19.1585	*ZRT2*	4.75	In *zap1-*	Y	1 and 2	Low affinity zinc transporter	Zinc ion transport
orf19.3112	*ZRT1*	4.56	In *zap1-*	Y	1 and 2	High affininty zinc transporter	Zinc ion transport
orf19.5585	*SAP5*	3.57	—	N	—	Secreted aspartyl proteinase 5	Biological adhesion
orf19.4477	*CSH1*	2.82	In *zap1-*	Y	2	Aryl-alcohol dehydrogenase	Cellular aldehyde metabolism
orf19.1048	*IFD6*	2.81	In *zap1-*	Y	—	Conserved aryl-alcohol dehydrogenase	Cellular aldehyde metabolism
orf19.5716	*SAP4*	2.47	—	N	—	Secreted aspartyl proteinase 4	Biological adhesion
orf19.1534	*(ZRT3)*	2.35	—	Y	1 and 2	Vacuolar Zn-iron permease	Zinc ion transport
orf19.4975	*HYR1*	2.10	—	N	—	Hyphally regulated protein	Hyphally regulated gene
orf19.1327	*RBT1*	2.03	—	N	—	Repressed by Tup1, related to HWP1	Hyphally regulated gene
orf19.4884	*WOR1*	−1.03	—	N	—	Regulator of white-opaque switching	Transcriptional regulator
orf19.5178	*ERG5*	−1.11	—	N	—	Cytochrome P450	Ergosterol biosynthesis
orf19.4504	*(ADH4)*	−1.19	—	N	—	Alcohol dehydrogenase	Alcohol dehydrogenase activity
orf19.4438	*RME1*	−1.23	—	N	—	Zinc-finger transcription factor	Transcriptional regulator
orf19.2608	*ADH5*	−1.31	In WT	N	—	Alcohol dehydrogenase II	Alcohol dehydrogenase activity
orf19.406	*ERG1*	−1.35	—	N	—	Squalene epoxidase (monooxygenase), ergosterol biosynthesis	Ergosterol biosynthesis
orf19.767	*ERG3*	−1.79	—	N	—	C-5 sterol desaturase	Ergosterol biosynthesis
orf19.999	*GCA2*	−1.80	In WT	N	—	Glucoamylase	Glucoamylase
orf19.3668	*HGT2*	−2.14	In WT	N	—	Hexose transporter	Carbohydrate transport
orf19.4899	*GCA1*	−2.43	In WT	N	—	Glycoamylase involved in beta-1,6-glucan synthesis;glucosidase II	Glucoamylase
orf19.4384	*HXT5*	−2.80	In WT	N	—	Fructose symporter	Carbohydrate transport
orf19.3618	*YWP1*	−3.25	In WT	N	—	Putative cell wall protein	Cell wall protein
orf19.3499	—	−3.33	In WT	N	—	Hypothetical protein	Function unknown
orf19.7094	*HGT12*	−4.54	—	N	—	Glucose sensor or transporter protein	Carbohydrate transport

a Systematic names and gene names are from the *Candida* Genome Database (http://www.candidagenome.org/.) Names in parentheses are recommendations.

b log2 Expression ratios are taken from our comparison of expression in the *zap1*Δ*/zap1*Δ*+*p*ZAP1* complemented strain and the *zap1*Δ*/zap1*Δ mutant (Dataset S4).

c This column indicates which host strain, carrying a *TDH3* promoter fusion for the respective gene, was characterized.

d This column indicates if the expression array target gene was also a direct binding target identified by ChIP–chip analysis. See Dataset S6 for the complete list of ChIP–chip target genes.

e This column indicates which of the two motifs determined by MEME analysis of the Chip–chip data are present in the 5′ promoter region of the target genes listed in this table. See Dataset S6 for the complete list of targets containing the two motifs. Motif 1 is ACCTTGGTGGTTA and Motif 2 is TAGTGGTTAT.

f GO terms are from the *Candida* Genome Database; other descriptors were coined for the sake of presentation.

Abbreviations: GO, gene ontology; N, no; WT, wild type; Y, yes.

**Figure 4:**
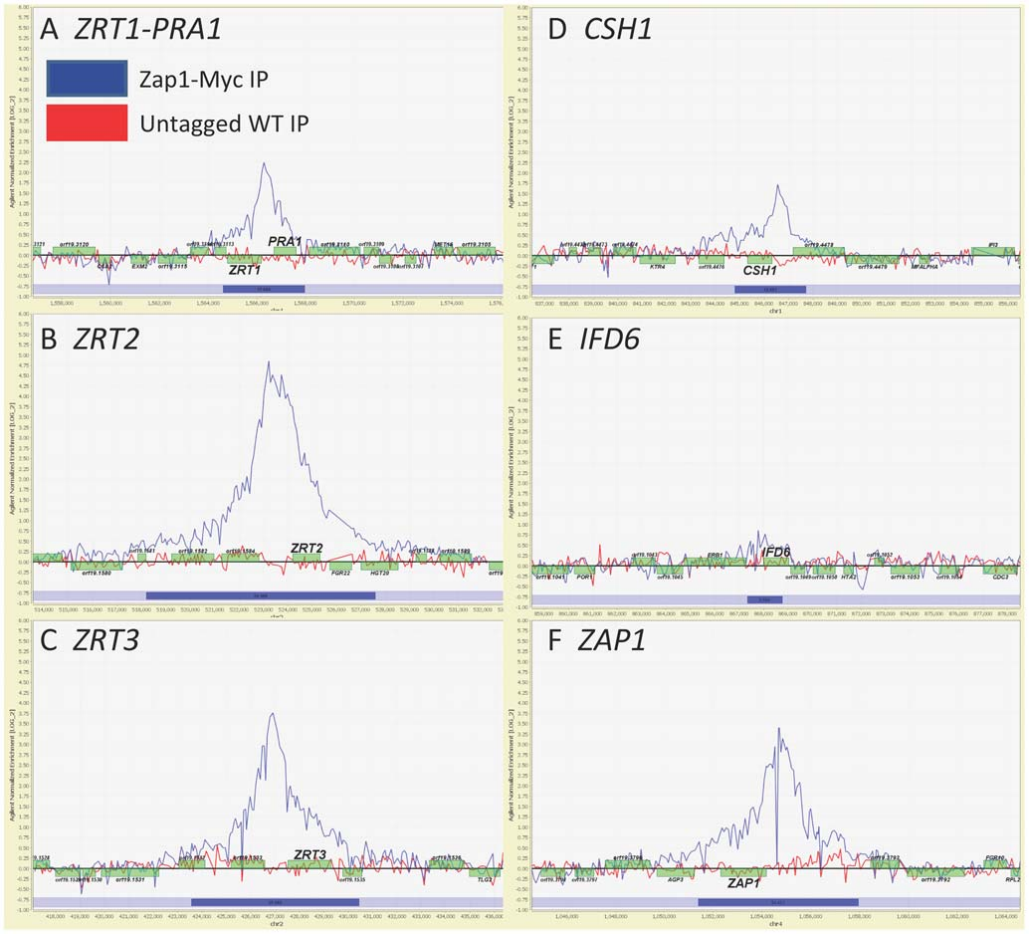
ChIP mapping of genomic Zap1 binding sites. Zap1 myc-tagged strain CJN1688 versus untagged wild-type strain DAY185 immunoprecipitation binding data were performed under biofilm conditions. The *x*-axis represents ORF chromosomal locations (See Dataset S6, sheet 1 for exact location values). The *y*-axis is the Agilent normalized enrichment value (log2) for binding of Zap1 (See Dataset S6, sheet 1 for exact enrichment values). Zap1-myc strain (blue line) and untagged wild-type (red line) ChIP–chip array binding data were mapped and plotted onto the chromosomes containing *ZRT1* and *PRA1* located on Chromosome 4 (A), *ZRT2* located on Chromosome 2 (B), *ZRT3* located on Chromosome 2 (C), *CSH1* located on Chromosome 1 (D), *IFD6* located on Chromosome 1 (E), and itself *ZAP1* located on Chromosome 4 (F) using ChipView v0.954. The promoters of these genes show significant peak enrichment (determined using Agilent Chip Analytics software v1.2) for the binding of Zap1. The blue track under the peak indicates that the Agilent segment *p*-value (−log10) for the binding of Zap1 is significant (See Dataset S6, sheet 1 for actual segment *p*-values). Genes plotted above the bold line read in the sense direction; genes plotted below the bold line read in the antisense direction. Identical binding sites with similar peak enrichment values were observed for the independently isolated Zap1 myc-tagged strain CJN1694 versus untagged wild-type strain DAY185 (unpublished data).

To identify target genes that are directly regulated by Zap1, we used genome-wide chromatin immunoprecipitation (ChIP) analysis of biofilm cells (Figure 4; Dataset S6). We found that Zap1 binds directly to the promoters of *ZRT1*, *ZRT2*, and *ZRT3* (Figure 4A–4C; Dataset S6), thus arguing that Zap1 regulates zinc homeostasis through activation of zinc transporter gene expression. The *ZRT1* 5′ region is shared with the divergent *PRA1* gene, whose *S. cerevisiae* ortholog *ZPS1* is a Zap1 target, so this shared regulatory region may permit Zap1 activation of both *ZRT1* and *PRA1* (Figure 4B). We also found Zap1 associated with its own (*ZAP1*) promoter region, as expected if *C. albicans* Zap1 activates its own expression (Figure 4F). We note that Zap1 autoregulation is well established in *S. cerevisiae* [15]. Finally, we found Zap1 bound to the promoters of *CSH1* and *IFD6* (Figure 4D, 4E), whose contribution to biofilm matrix is described below. Although *S. cerevisiae* Zap1 can function as a repressor [16], we did not detect *C. albicans* Zap1 bound to promoter regions of genes identified by microarrays to be repressed including *ADH5*, *GCA1*, or *GCA2*. (ChipView plots of every significant binding event may be found in Dataset S6, sheet 3.) These genes may be indirectly regulated by Zap1. It is also formally possible that Zap1 associates with other proteins that mask the epitope in order to function as a repressor; according to this model we would fail to detect genes where Zap1 was bound as a repressor. Overall, our data clearly show that Zap1 directly activates many target genes that function in diverse biological processes.

### Function of Zap1 Target Genes in Biofilm Matrix Production

**Figure 5:**
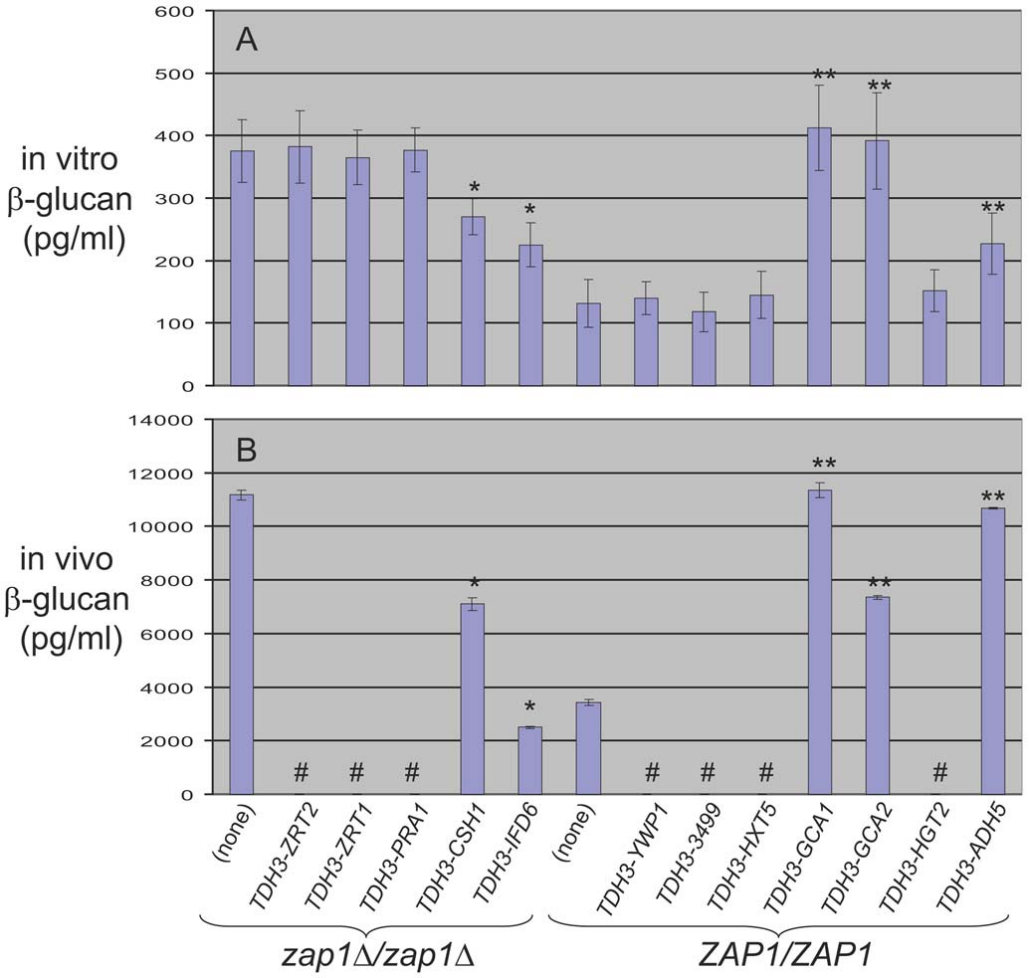
Effect of altered Zap1 target gene expression. Soluble β-1,3 glucan levels were determined after biofilm growth (A) in vitro or (B) in the rat catheter model. Determinations were carried out with *zap1*Δ*/zap1*Δ strains carrying either no promoter fusion or *TDH3* promoter fusions to genes *ZRT2*, *ZRT1*, *PRA1*, *CSH1*, or *IFD6*, as indicated in the figure. Determinations were also carried out with *ZAP1/ZAP1* strains carrying either no promoter fusion, or *TDH3* promoter fusions to genes *YWP1*, *orf19.3499*, *HXT5*, *GCA1*, *GCA2*, *HGT2*, or *ADH5*, as indicated in the figure. A single asterisk indicates that glucan measurements were significantly different (*p*<0.05) from the *zap1*Δ*/zap1*Δ strain carrying no promoter fusion; a double asterisk indicates that glucan measurements were significantly different (*p*<0.05) from the *ZAP1/ZAP1* strain carrying no promoter fusion; both assessments are based upon Student's *t*-tests. In (B), the pound symbol (#) indicates that the respective strain was not assayed in the in vivo biofilm model.

We further investigated several Zap1 target genes that may function in biofilm matrix production (Table 1). Genes that are downregulated in the *zap1*Δ*/zap1*Δ mutant could, in principle, be inhibitors of matrix production; genes that are upregulated in the *zap1*Δ*/zap1*Δ mutant could be activators of matrix production. We reasoned that overexpression of matrix inhibitors in the *zap1*Δ*/zap1*Δ mutant may cause reduced levels of soluble β-1,3 glucan. To test this idea, we introduced highly expressed *TDH3* promoter sequences to replace promoter regions of the following target genes: *ZRT2*, *ZRT1*, *PRA1*, *CSH1*, and *IFD6.* We confirmed their overexpression through qPCR assays in the *zap1*Δ*/zap1*Δ transformants (Dataset S5). We observed that both *TDH3-CSH1* and *TDH3-IFD6* caused a significant decrease in soluble β-1,3 glucan levels produced by in vitro biofilms (Figure 5A), whereas the other constructs produced no significant differences. To survey candidate activators of matrix production, we overexpressed selected genes in a wild-type (*ZAP1/ZAP1*) background. Once again, we used the *TDH3* promoter to replace promoter regions of target genes *YWP1*, *orf19.3499*, *HXT5*, *GCA1*, *GCA2*, *HGT2*, and *ADH5*, and used qPCR to confirm overexpression (Dataset S5). We observed that *TDH3-GCA1*, *TDH3-GCA2*, and *TDH3-ADH5*, but not the other constructs, significantly increased soluble β-1,3 glucan levels produced by in vitro biofilms (Figure 5A). These results support the idea that specific Zap1 target genes can modulate biofilm matrix levels in vitro.

To test target gene function in vivo, we turned to the rat catheter infection model. We measured biofilm-associated soluble β-1,3 glucan levels after biofilm formation by the strains that had displayed altered glucan levels in vitro. The general effects on soluble β-1,3 glucan of each *TDH3-*target gene during biofilm culture in vivo paralleled those measured in vitro (Figure 5B), though the magnitudes of the effects were typically greater in vivo. These findings indicate that Csh1 and Ifd6 are inhibitors of matrix production, and that Gca1, Gca2, and Adh5 are activators of matrix production.

## Discussion

Matrix is a defining characteristic of biofilms [3]–[6], and has been found to contribute, in many organisms, to such critical biofilm attributes as adherence and antimicrobial drug resistance. The matrix of *C. albicans* biofilms has been characterized biochemically [8],[17], but its biogenesis and regulation have remained elusive. We report here that *C. albicans* Zap1 governs biogenesis of a major matrix component, soluble β-1,3 glucan. Our characterization of the Zap1 regulon, together with recent studies by Kim and colleagues [11], confirms the functional conservation of Zap1 as a regulator of zinc metabolism. We show that, in *C. albicans*, the Zap1 regulon extends to govern both positive and negative matrix biogenesis functions, and identification of key Zap1-regulated genes gives insight into the metabolic processes that contribute to biofilm formation. Based on the relationship between Zap1 and matrix, as well as other Zap1 target genes, it is likely that Zap1 functions broadly as a negative regulator of biofilm maturation.

### Zap1-Responsive Genes

*C. albicans* Zap1, like its *S. cerevisiae* ortholog, has a critical role in zinc metabolism. Genes activated by *C. albicans* Zap1 include putative plasma membrane zinc transporter genes *ZRT1* and *ZRT2* as well as the putative vacuolar zinc transporter gene *ZRT3*. Both homology and functional analysis indicates that these genes are connected to zinc acquisition ([11] and this report). Thus the connection of Zap1 to zinc metabolism is clear.

**Figure 6:**
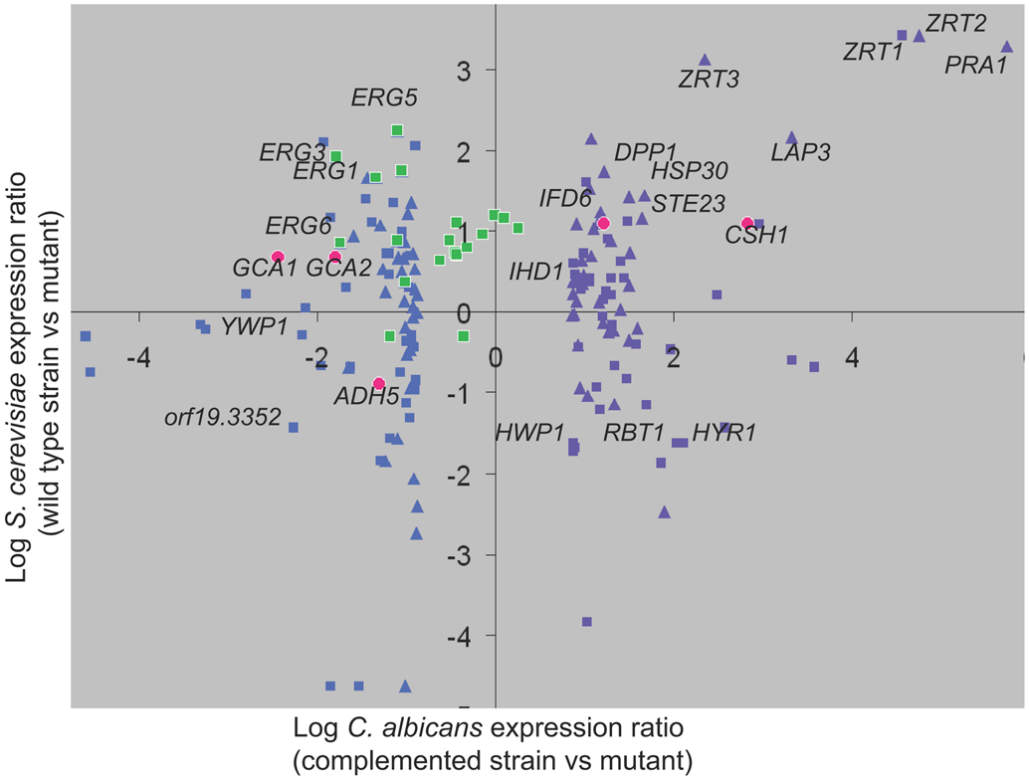
Comparison of *C. albicans* and *S. cerevisiae* Zap1 regulons. Expression of Zap1-responsive genes in *C. albicans* (complemented strain versus *zap1*Δ*/zap1*Δ mutant, *x*-axis) was compared with their *S. cerevisiae* orthologs and best hits (wild-type strain versus *zap1* mutant, *y*-axis). Definitions of orthologous genes and best hits were provided by the *Candida* Genome Database (see Dataset S4; worksheet 3; (http://www.candidagenome.org/download/homology/orthologs/Calb_Scer_by_inparanoid/Assem21orthologs/CA_SC_orthologs.txt and http://www.candidagenome.org/download/homology/best_hits/Calb_Scer_best_hits_Assem21.txt). Expression data for *S. cerevisiae* were for growth in 61 nM zinc from Lyons et al. [18]. This graph presents the 40 most downregulated genes (purple triangles) and 40 most upregulated genes (blue triangles) in the *zap1*Δ*/zal1*Δ mutant compared to *S. cerevisiae* orthologs, and the 40 most downregulated genes (purple squares) and 40 most upregulated genes (blue squares) in the *zap1*Δ*/zal1*Δ mutant compared to *S. cerevisiae* best hits. In addition, all *C. albicans ERG* genes are graphed against their orthologs or best hits (green squares). Finally, the five genes shown to be functionally relevant for biofilm matrix are graphed against their orthologs or best hits (red circles).

Interestingly, the conserved Zap1 circuit encompasses many additional genes, as indicated by comparison of Zap1-responsive genes in our dataset with their *S. cerevisiae* orthologs and best hits [18]. Conserved Zap1-responsive genes extend beyond zinc transporter genes (Figure 6; Dataset S4, worksheet 3) to include such Zap1-activated genes as *PRA1*, *DPP1*, *HSP30*, *LAP3*, *STE23*, *CSH1*, and *IFD6.* Conserved Zap1-repressed genes include *ADH5* and *orf19.3352*, among many more (Figure 6). The extent of conservation may be underestimated because of the different growth conditions employed for the two organisms, and the fact that the *S. cerevisiae* Zap1 regulon varies with conditions of zinc limitation [19]. Some of these gene products are known or predicted to be zinc metalloenzymes, such as Ste23, and their increased expression in *zap1* mutants may reflect a homeostatic response to reduced enzyme activity. However, the relationship of many of conserved Zap1-dependent genes to zinc acquisition or metabolism is not well understood. We note in particular that the secreted metalloprotease homolog Pra1, the ortholog of *S. cerevisiae* Zps1, is also closely related to the *Aspergillus fumigatus* antigen ASPF2 (44% identity over 294 amino acid residues), which is induced under low zinc conditions [20]. Thus the Zap1 regulon may be broadly conserved among fungi. Genes with conserved Zap1 responsive regulation in fungi with distinct environmental niches might be considered priorities for further study in relationship to zinc metabolism. Conversely, species-specific responses may provide insight into unique features of each zinc-limited niche.

MEME analysis of direct Zap1 target genes has identified two potential Zap1 binding motifs, ACCTTGGTGGTTA and TAGTGGTTAT (motifs 1 and 2, respectively, in Dataset S6, worksheet 2), which are similar to each other. RSAT analysis points to enriched 8-mers TAATGGTG and ATGGTGGT in these 5′ regions, which closely resemble the MEME sites. All are similar to the known *S. cerevisiae* Zap1 binding motif, ACCTTNAAGGT [21],[22], particularly because the greatest specificity is for the motif ends ACC and GGT [23].

*C. albicans* biofilm growth is associated with overall upregulation of ergosterol biosynthesis [24] as well as increased resistance to antifungals that target ergosterol [7],[25]. It is striking that almost all ergosterol biosynthetic genes are regulated oppositely by Zap1 in *C. albicans* and *S. cerevisiae* (Figure 6 [green squares]; Dataset S4, worksheet 3). *ERG* genes are largely downregulated in the *S. cerevisiae zap1*Δ mutant; in other words, ScZap1 is formally a positive regulator of *ScERG* genes. This relationship has functional consequences, because a *S. cerevisiae zap1*Δ*/ZAP1* heterozygous diploid is hypersensitive to ergosterol biosynthetic inhibitors [26]. In contrast, *ERG* genes are largely upregulated in the *C. albicans zap1*Δ*/zap1*Δ mutant, thus CaZap1 is formally a negative regulator of *CaERG* genes. Zap1 may govern their expression indirectly, because they lack clear ZREs and were not bound by Zap1 in our ChIP analysis. This difference in *ERG* gene regulation may reflect the distinct niches sampled for microarray analysis: *S. cerevisiae* cells were grown aerobically [18]; our *C. albicans* cells were grown in biofilms, which are substantially anaerobic [27]. It is well established that *ERG* gene expression responds to oxygen levels [28], a reflection of the heme requirement for ergosterol synthesis. The apparently opposite roles of Zap1 in *ERG* gene regulation in the two organisms may arise from the difference in growth conditions. In any event, for *C. albicans* biofilms, perhaps a decline in Zap1 activity during biofilm growth may be the cause of increased ergosterol biosynthetic gene expression in biofilms.

### Biofilm Matrix Synthesis

In principle, Zap1 might have influenced matrix production indirectly, as a consequence of poor growth or zinc limitation. However, overexpression of *ZRT1* or *ZRT2* improves zinc-limited growth of the *zap1*Δ*/zap1*Δ mutant but has no effect on matrix production. These findings indicate that it is altered Zap1 target gene expression, rather than other effects of zinc limitation, that stimulates matrix production in the *zap1*Δ*/zap1*Δ mutant. Our target gene overexpression studies point to two classes of matrix biogenesis functions: Csh1 and Ifd6 inhibit matrix production; Gca1, Gca2, and Adh5 promote matrix production.

The role of Gca1 and Gca2 in matrix production is probably direct. They are predicted extracellular glucoamylases; the extracellular localization of Gca1 has been confirmed by biochemical isolation [29]. Glucoamylases convert long-chain polysaccharides into smaller-chain polysaccharides. Therefore, we propose that Gca1 and Gca2 promote matrix production by hydrolytic release of soluble β-1,3 glucan fragments, perhaps from biofilm cell walls, from exported glucan polymers that are not attached to cell walls, or from debris of lysed cells.

The roles of Csh1, Ifd6, and Adh5 may be more complex. All three are predicted alcohol dehydrogenases. One simple possibility is that they affect matrix production through their impact on carbon metabolism. For example, Adh5 may promote entry of ethanol into the TCA cycle for energy or via the glyoxylate shunt to provide hexose for β-1,3 glucan synthesis. Ethanol is known to accumulate in mature biofilms [30] and thus may serve as a potential source of carbon. However, this explanation does not readily account for the fact that Adh5 stimulates matrix production, whereas Csh1 and Ifd6 inhibit matrix production. A second model is based upon the roles of alcohol dehydrogenases in the Ehrlich pathway [31]. This pathway permits nitrogen assimilation from amino acids, yielding α-keto acids that must be reduced to acyl and aryl alcohols for secretion. Such alcohols have profound roles in quorum sensing and cell signaling. One aryl alcohol, tyrosol, accumulates during biofilm maturation and functions to stimulate hyphal growth [32],[33]. The acyl alcohol farnesol also accumulates during biofilm maturation [34] and inhibits hyphal growth and biofilm formation [35]–[37]. Additional complex alcohols that inhibit hyphal growth also accumulate in *C. albicans* biofilms during maturation [34]. With these studies as backdrop, a simple model is that Csh1, Ifd6, and Adh5 catalyze the final reductive step in the biogenesis of biofilm-associated acyl and aryl alcohols, and these alcohols act as signals to govern matrix synthesis. The apparently opposite effects of these gene products on matrix production may be related to substrate specificity: Csh1 and Ifd6 may act preferentially to yield a matrix inhibitory signal; Adh5 may act preferentially to yield a matrix stimulatory signal.

The idea that Zap1 governs quorum-sensing molecule synthesis explains the unexpected cell morphology observed in *zap1*Δ*/zap1*Δ mutant biofilms. Specifically, we observed an excess of yeast-form cells along with some unusually round cells that resemble chlamydospores. Consistent with the apparent accumulation of yeast-form cells, we note that the *zap1*Δ*/zap1*Δ mutant shows upregulation of yeast-specific gene *YWP1* and downregulation of hyphally induced genes *HWP1*, *RBT1*, *HYR1*, and *IHD1* (Figure 6). Growth of yeast-form cells and chlamydospores is promoted by the quorum-sensing molecule farnesol [35],[38],[39]. However, there has been thus far no clear connection between quorum-sensing molecules and biofilm matrix. Although this connection is speculative at present, we note that it makes testable predictions; in particular, that accumulation of specific acyl and aryl alcohols will be modulated by Zap1 and by these alcohol dehydrogenases. Similarly, it predicts that other defects in biogenesis of Ehrlich pathway precursors will modulate matrix production.

The unexpected connection of *C. albicans* Zap1 to matrix production raises the question of whether the relevant target genes are part of the conserved Zap1 regulon. We find that three of the genes are (Figure 6): *C. albicans CSH1* and *IFD6* share the *S. cerevisiae* best hit YPL088W; *C. albicans ADH5* has the *S. cerevisiae* best hit *ADH5.* All of these genes are under Zap1 control in the respective organisms. On the other hand, *GCA1* and *GCA2* share the *S. cerevisiae* best hit *ROT2*, which is not significantly responsive to *S. cerevisiae* Zap1 under conditions examined [18]. These findings indicate that a focus limited either to conserved or novel Zap1-responsive genes would have revealed some functional targets and overlooked others.

### Integration of Zap1 Activity into *C. albicans* Biofilm Formation

**Figure 7:**
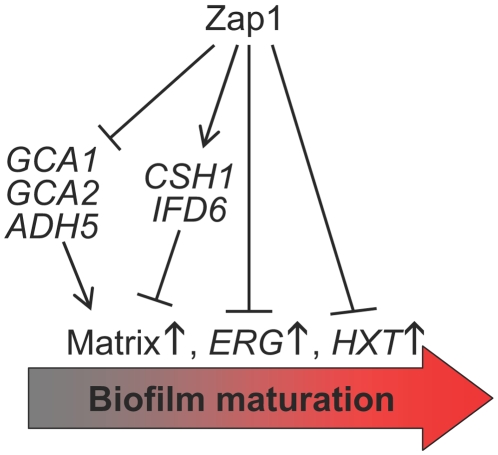
Integration of Zap1 function into biofilm formation. Zap1 functions as a negative regulator of biofilm matrix accumulation. It does so through activation of expression of *CSH1* and *IFD6*, which inhibit matrix accumulation, and through repression of expression of *GCA1*, *GCA2*, and *ADH5*, which promote matrix accumulation. Zap1 binds to the *CSH1* and *IFD6* promoter regions and thus is likely to activate their expression directly. Zap1 is a negative regulator of two gene classes—ERG genes and *HXT* genes—that that are upregulated during biofilm development [24]. We suggest that Zap1 functions as a negative regulator of several aspects of biofilm maturation.

The *zap1*Δ*/zap1*Δ mutant produces a biofilm with exaggerated features of mature biofilms. We have focused here on the abundance of matrix, but there are other such features as well. For example, the mutant biofilm hyphal layer includes an apparent excess of yeast-form cells, which may be induced in mature biofilms by accumulation of quorum-sensing molecules [4],[32],[34] to facilitate biofilm dispersal. The upregulation of *ERG* genes and hexose transporter genes in the mutant are other features in common with mature biofilms [24]. A simple working hypothesis is that Zap1 functions as a negative regulator of biofilm maturation (Figure 7). We suggest that a decline in Zap1 activity during biofilm development may occur during the natural process of biofilm maturation to bring about these characteristic biological features.

## Material and Methods

### Media

*C. albicans* strains were grown at 30°C in either YPD (2% Bacto peptone, 2% dextrose, 1% yeast extract) for Ura+ strains or in YPD+uri (2% Bacto peptone, 2% dextrose, 1% yeast extract, and 80 µg/ml uridine) for Ura− strains. Transformants were selected for on synthetic medium (2% dextrose, 6.7% Difco yeast nitrogen base with ammonium sulfate and auxotrophic supplements) or on YPD+clonNAT400 (2% Bacto peptone, 2% dextrose, 1% yeast extract, and 400 µg/ml nourseothricin [clonNAT, WERNER BioAgents]) for nourseothricin-resistant isolates. Growth on low-zinc medium was assayed with synthetic medium lacking added zinc (2% dextrose, 1.7% yeast nitrogen base without ammonium sulfate and without zinc sulfate, 0.2% ammonium sulfate, 2.5 µM EDTA, and auxotrophic supplements). To obtain nourseothricin-sensitive isolates having flipped out the *SAT1* marker, nourseothricin-resistant transformants were grown for 8–12 h in YPD liquid medium, plated at a low cell density of 200 cells/plate on YPD+clonNat25 (2% Bacto peptone, 2% dextrose, 1% yeast extract, and 25 µg/ml nourseothricin [clonNAT, WERNER BioAgents]), and allowed to grow for 24 h at 30°C as previously described [40] with the defined modifications. Biofilms for visualization were grown using Spider medium [41]. Supernatants collected for β-1,3 glucan measurements were grown in suspension or as biofilms in RPMI-MOPS medium for 12 h at 37°C, as described previously [10].

### Plasmid and Strain Construction

All *C. albicans* strains used in this study are listed in Dataset S1. Reference strain DAY185 has been described [42]. Newly constructed *C. albicans* strains were derived from BWP17 [43]. Primer sequences are listed in Dataset S2. All genotypes were verified by colony PCR using corresponding detection primers (Dataset S2). Construction of CJN1091 (*zap1/zap1*) was made by PCR product-directed gene deletion [43] with 120-mer oligonucleotides CSR1null-5DR and CSR1null-3DR via consecutive rounds of transformation into BWP17. For gene complementation, PCR was used to generate a fragment for *ZAP1* from 1,000 bp upstream of the start codon to 500 bp downstream of the stop codon. This fragment was inserted into pGEMT-Easy (Promega), digested with NgoMIV and AlwNI, and subsequently inserted by in vivo recombination in *S. cerevisiae* into NotI- and EcoRI-digested *HIS1* vector pDDB78 [44], yielding plasmid pCJN517. The complemented strain CJN1193 was made by transforming CJN1091 with NruI-digested pCJN517, directing integration to the *HIS1* locus. The *zap1/zap1* mutant strain was made His+ by transforming CJN1091 with NruI-digested pDDB78 to yield strain CJN1201.

The *NAT1-TDH3* promoter plasmid pCJN542 [45] was used for gene overexpression. The *TDH3-IFD4* overexpression strain CJN1680 was constructed by transforming CJN1201, the *zap1/zap1* mutant, using PCR products from template plasmid pCJN542 and primers IFD4-F-OE-Ag-NAT-Ag-p-CJN and IFD4-R-OE-Ag-NAT-Ag-TDH3p-CJN. These primers amplify the entire *Ashbya gossypii TEF1* promoter, the *C. albicans NAT1* open reading frame, the *A. gossypii TEF1* terminator, and the *C. albicans TDH3* promoter with 100 bp of hanging homology to 500 bp upstream into the promoter of *IFD4* for the forward primer and 100 bp of hanging homology from exactly the start codon of *IFD4*. The homology in these primers allows for homologous recombination of the entire cassette directly upstream of the natural locus of *IFD4* so that its expression is driven by the *TDH3* promoter instead of its natural promoter. By the same method, primers IFD6-F-OE-Ag-NAT-Ag-p-CJN and IFD6-R-OE-Ag-NAT-Ag-TDH3p-CJN were used for overexpression of *IFD6* to produce strain CJN1631; ZRT2-F-OE-Ag-NAT-Ag-TEF1p and ZRT2-R-OE-Ag-NAT-Ag-TDH3p-CJN for overexpression of *ZRT2* to produce strain CJN1655; ZRT1-F-OE-Ag-NAT-Ag-TEF1p-CJN and ZRT1-R-OE-Ag-NAT-Ag-TDH3p-CJN for overexpression of *ZRT1* to produce strain CJN1651; and PRA1-F-OE-Ag-NAT-Ag-p-CJN and PRA1-R-OE-Ag-NAT-Ag-TDH3p-CJN for overexpression of *PRA1* to produce strain CJN1623. The *TDH3-19.4899* overexpression strain CJN1638 was constructed by transforming DAY185, the wild-type reference strain, using PCR products from template plasmid pCJN542 and primers 4899-F-OE-Ag-NAT-Ag-p-CJN and 4899-R-OE-Ag-NAT-Ag-TDH3p-CJN. By the same method, primers 999-F-OE-Ag-NAT-Ag-p-CJN and 999-R-OE-Ag-NAT-Ag-TDH3p-CJN were used for overexpression of *ORF19.999* to produce strain CJN1675; ADH5-F-OE-Ag-NAT-Ag-p-CJN and ADH5-R-OE-Ag-NAT-Ag-TDH3p-CJN for overexpression of *ADH5* to produce strain CJN1642; YWP1-F-OE-Ag-NAT-Ag-p-CJN and YWP1-R-OE-Ag-NAT-Ag-TDH3p-CJN for overexpression of *YWP1* to produce strain CJN1659; 3499-F-OE-Ag-NAT-Ag-p-CJN and 3499-R-OE-Ag-NAT-Ag-TDH3p-CJN for overexpression of *ORF19.3499* to produce strain CJN1633; 4384-F-OE-Ag-NAT-Ag-p-CJN and 4384-R-OE-Ag-NAT-Ag-TDH3p-CJN for overexpression of *HXT5* to produce strain CJN1663; and HGT2-F-OE-Ag-NAT-Ag-p-CJN and HGT2-R-OE-Ag-NAT-Ag-TDH3p-CJN for overexpression of *HGT2* to produce strain CJN1667. Transformation into *C. albicans* strains and selection on YPD+clonNAT400 plates has been described [46]. Integration of the constructs was verified by colony PCR with a gene-specific forward detection primer (for example primer IFD4-OE-F-det-CJN for the *IFD4* gene), annealing to a sequence within the promoter of each gene and the reverse primer Nat-OE-R-det2-CJN annealing to a sequence found in the *NAT* gene.

The C-terminal myc-tagging plasmid pADH34 (Dataset S3), containing a 13myc epitope tag immediately preceding the *SAT1*-flipper cassette (34-bp *FLP* recombination target sequence [*FRT*], followed by the *C. albicans MAL2* promoter, followed by a *C. albicans*-adapted *FLP* gene, followed by a *C. albicans ACT1* terminator sequence, followed by the *C. albicans*-adapted *SAT1* marker gene, followed by another 34-bp *FRT* sequence), was constructed as follows. PCR was done using template pFA6a-13myc-kanMX6 [47] and primers AHO276 and AHO277 to generate a 568-bp product containing a 13myc epitope tag and linker sequences with flanking XhoI sites. This fragment was ligated into the unique XhoI site of the *SAT1*-flipper cassette plasmid, pSFS2A [40], yielding plasmid pADH34. The C-terminal tagged nourseothricin-resistant Zap1-myc strains, CJN1684 and CJN1685, were constructed by transforming DAY185, the reference strain, using PCR products from template plasmid pADH34 and primers 3794MycFnostop-CJN and 3794MycRUTR-CJN. These primers amplify the entire 13myc epitope tag and complete *SAT1* flipper cassette with 65 bp of hanging homology to the *ZAP1* ORF minus its stop codon for the forward primer and 65 bp of hanging homology to the *ZAP1* UTR precisely downstream of the stop codon for the reverse primer. The homology in these primers allows recombination of the entire 13myc epitope tag and complete *SAT1* flipper cassette directly downstream of the *ZAP1* ORF, lacking its natural stop codon, so that the *ZAP1* ORF contains a C-terminal 13myc epitope tag translational fusion. Correct integration of the C-terminal 13myc epitope tag and *SAT1* flipper was verified by colony PCR using detection primers 3794detFUpMyctag-CJN and AHO300 to check the upstream integration and 3794detRDownMyctag-CJN and AHO301 to check the downstream integration. The C-terminal tagged nourseothricin-sensitive Zap1-myc strains, CJN1688 and CJN1694, were constructed by flipping out the *SAT1* cassette from strains CJN1684 and CJN1685, respectively, as described previously [40]. The following primer pairs were used in colony PCR to confirm the clean “flipping out” of the *SAT1*-flipper cassette: 3794detFUpMyctag-CJN and AHO300, and 3794detRDownMyctag-CJN and AHO302. The 13myc epitope tag and the region of homology to the 3′ end of *ZAP1* used for integration of the *SAT1*-flipper cassette was confirmed by sequencing the colony PCR product generated using primers 3794detFUpMyctag-CJN and AHO283.

### In Vitro Biofilm Growth, Microscopy, and Biomass Determination

In vitro biofilm growth assays were carried out in Spider medium and visualized by CSLM as described previously [12]. Biomass measurements were determined for four independent silicone samples as described previously [46].

### In Vivo Biofilm Model

A rat central-venous-catheter infection model, as described previously [14], was selected for our in vivo biofilm studies. We removed catheters from the rats at 24 h after *C. albicans* infection to determine biofilm development on the internal surface of the intravascular devices. The distal 2 cm of the catheter was cut from the entire catheter length, and biofilms were imaged by SEM at 50× and 1,000× magnification, as described previously [9].

### Secreted β-1,3 Glucan Measurements from Biofilm and Planktonic Growth In Vitro

Cultures were grown on silicone disks or in suspension in RPMI medium, as described above. Culture supernatants from *C. albicans* in vitro biofilm and planktonic cells were collected at 12 h for glucan measurements. Viable cell burdens were determined using plate counts to ensure the cultures contained similar number of cells. Supernatants were centrifuged at 3,000*g* for 10 min, and were stored at −20°C until glucan analysis. Glucan concentrations were determined using the commercially available Glucatell (1,3)-β-D-Glucan Detection Reagent kit (Associates of Cape Cod) according to manufacturer's directions. Four in vitro glucan assay replicates were performed for each sample. Statistical significance (*p*-values) was determined with a Student's *t*-test.

### Secreted β-1,3 Glucan Measurements from Biofilm Growth In Vivo

After 12 h of growth in the in vivo biofilm model, serum was collected from the venous catheter. Serum samples were frozen at −20°C until glucan analysis. β-1,3 glucan was measured in the serum using the Fungitell (1,3)-β-D-Glucan Detection Reagent kit (Associates of Cape Cod) according to manufacturer's directions. Three in vivo glucan assay replicates were performed for each rat catheter. Statistical significance (*p*-values) was determined with a Student's *t*-test. Viable cell burdens were measured by harvesting kidneys at the end of the experiment as an estimation of total-body organ burden.

### RNA Extraction from Biofilms

Biofilms for expression microarray analysis were grown in Spider medium at 37°C without silicone squares. Instead, the bottom of a six-well polystyrene plate was used as a substrate for biofilm growth in order to maximize the efficiency of harvesting cells for RNA extraction. We find that one six-well plate containing biofilms for one strain yields sufficient RNA for expression microarray analysis. Similar to the silicone square method [12], the bottom of the six-well plates were pretreated overnight in 4 ml bovine serum (Gibco), and placed at 37°C with 200-rpm agitation in a thermostatic Elmi shaker. Concurrently, standard overnight cultures of the strains of interest were inoculated in YPD medium at 30°C with shaking. The following day, the six-well plates were washed with PBS, 4 ml Spider medium was added to each well, and the overnight culture was added to each well in order to obtain a starting OD_600_ in the 4 ml Spider well volume of 0.5. Cell adherence was done for 90 min by placing the six-well plates at 37°C with 200-rpm agitation in the Elmi shaker. After the cell adherence step, the six-well plates were washed with PBS, and 4 ml of fresh Spider medium was added to the wells. Biofilms were grown for 48 h at 37°C with 200-rpm agitation in the Elmi shaker. Biofilms were harvested by scraping the bottoms of the six-well plates with a cell scraper, and combining the biofilm slurry of the same strain from each well of one six-well plate in a 50-ml conical tube. Biofilm cells were then centrifuged at 3,000*g* for 5 min, and RNA was extracted using the RiboPure-Yeast RNA kit (Ambion, number AM1926) according to the manufacturer's instruction. We find that this kit yields the cleanest, most stable, and highest quality and quantity of RNA compared with the hot phenol method for extraction of RNA from a *C. albicans* biofilm.

### Northern and Quantitative PCR Expression Analysis

Northern analysis was performed as described previously [12] to verify the expression levels of *ZAP1*, *ZRT2*, and *ZRT1* using the primers ZAP1-FNor and ZAP1-RNor for *ZAP1*, ZRT2-FNor and ZRT2-RNor for *ZRT2*, and ZRT1-FNor and ZRT1-RNor for *ZRT1*. For quantitative real-time reverse transcription-PCR (qPCR) analysis, 10 µg of total RNA was DNase-treated at 37°C for 1 h using the DNA-free kit (Ambion), cDNA was synthesized using the AffinityScript multiple temperature cDNA synthesis kit (Stratagene), and qPCR was done using the iQ SYBR Green Supermix (Bio-Rad) as previously described [45] using the primers ZRT2-FqRTPCR and ZRT2-RqRTPCR for *ZRT2*, ZRT1-FqRTPCR and ZRT1-RqRTPCR for *ZRT1*, PRA1-FqRTPCR and PRA1-RqRTPCR for *PRA1*, IFD4-FqRTPCR and IFD4-RqRTPCR for *IFD4*, IFD6-FqRTPCR and IFD6-RqRTPCR for *IFD6*, ZAP1-FqRTPCR and ZAP1-RqRTPCR for *ZAP1*, YWP1-FqRTPCR and YWP1-RqRTPCR for *YWP1*, 3499-FqRTPCR and 3499-RqRTPCR for *ORF19.3499*, HXT5-FqRTPCR and HXT5-RqRTPCR for *HXT5*, 4899-FqRTPCR and 4899-RqRTPCR for *ORF19.4899*, 999-FqRTPCR and 999-RqRTPCR for *ORF19.999*, HGT2-FqRTPCR and HGT2-RqRTPCR for *HGT2*, and ADH5-FqRTPCR and ADH5-RqRTPCR for *ADH5*. The iCycler iQ detection system (Bio-Rad) was used with the following program: initial denaturation at 95°C for 5 min, followed by 40 cycles of 95°C for 45 s, 58°C for 30 s, and 72°C for 30 s. Amplification specificity was determined by melting curve analysis. Bio-Rad iQ5 software was used to calculate normalized gene expression values using the ΔΔCt method, using *TDH3* as a reference gene. For ease of interpretation, the reference strain expression level values were set to 1.0 for each gene set, and the normalized expression of each gene relative to *TDH3* expression is shown. Results are the means of three determinations.

### Expression Array Design and Analysis

Transcription expression profiling using long-oligonucleotide microarrays was performed as previously described [48]. Briefly, 10 µg of total biofilm RNA was DNase-treated at 37°C for 1 h using the DNA-free kit (Ambion), and cDNA was synthesized using the AffinityScript multiple temperature cDNA synthesis kit (Stratagene). We performed four individual hybridization experiments from four pairs of independently produced RNA samples of CJN1201, the *zap1/zap1* mutant strain versus CJN1193, the *zap1/zap1*+p*ZAP1* strain. LOWESS normalization and statistical analysis of the data were conducted in GeneSpring GX version 7.3 (Agilent Technologies). Data are reported in Dataset S4. A volcano-plot algorithm was used to identify genes that exhibited statistical significance (*p*<0.05) with a change in transcript abundance of at least 1.5-fold. The results of this analysis with adjusted *p*<0.05 are listed in Dataset S4 (worksheet 2).

### Full Genome ChIP Tiling Array (ChIP–chip)

The ChIP–chip tiling arrays were designed by tiling 181,900 probes of 60-bp length across 14.3 Mb included in the *C. albicans* Assembly 20 genome (http://www.candidagenome.org/), as previously described [49]. The Zap1 myc-tagged strains CJN1688 and CJN1694 and the untagged reference strain DAY185 were grown under the same biofilm-inducing conditions as the strains grown for expression microarray analysis, described above. We found that one six-well plate per strain yielded sufficient starting material to complete a single ChIP–chip experiment. Biofilms were harvested by scraping the bottoms of the six-well plates with a cell scraper, and combining the biofilm slurry of the same strain from each well of one six-well plate in a 50-ml conical tube. Formaldehyde was added to the biofilm slurry to a final concentration of 1%, and the treated biofilm cultures were mixed on a platform shaker for 15 min at room temperature. Glycine was then added to a final concentration of 125 mM, and the treated cultures were mixed for another 5 min at room temperature on the platform shaker. The following cell lysis and ChIP–chip methods were adapted from previously described protocols [49],[50]. Cells were collected by centrifugation at 4°C for 10 min at 3,000*g*, washed twice in 10 ml ice cold TBS (20 mM TrisHCl [pH 7.6], 150 mM NaCl), and the pellets frozen in liquid nitrogen prior to cell lysis. Cell lysis and shearing of DNA were done by resuspending the pellets in 700 µl lysis buffer (50 mM HEPES/KOH [pH 7.5], 140 mM NaCl, 1 mM EDTA, 1% Triton X-100, 0.1% Na-Deoxycholate) supplemented with complete protease inhibitor cocktail tablets (Roche). The cell suspension was vortexed at 4°C for 4 h in the presence of 0.5-mm acid-washed glass beads, and the lysate was collected. Chromatin was sheared by sonication in a Bioruptor water bath sonicator (settings: 1×15 min, 30 s on, 1 min off) at 4°C, the sheared lysate was centrifuged at 12,000*g* for 10 min at 4°C, and the supernatant was collected. 50 µl of extract was added to 200 µl TE/1% SDS, and stored at −20°C as the ChIP input material. For chromatin IPs, 300 µl of the crude lysate was added to 200 µl lysis buffer, and 10 µl of mouse monoclonal antihuman c-myc antibody (Biosource, number AHO0062) was added to the mixture. Extract plus antibody was incubated overnight at 4°C, with agitation. The following day, 50 µl of a 50% suspension of protein G-Sepharose Fast-Flow beads (Sigma) in lysis buffer was added and incubated 2 h at 4°C, with agitation. The beads were pelleted for 1 min at 1,000*g*, the supernatant removed, and the beads washed 5 min at room temperature with ice-cold buffers as follows: twice in lysis buffer, twice in high salt lysis buffer (50 mM HEPES-KOH [pH 7.5], 500 mM NaCl, 1 mM EDTA, 1% Triton X-100, 0.1% sodium deoxycholate), twice in wash buffer (10 mM Tris-HCl [pH 8.0], 250 mM LiCl, 0.5% NP-40, 0.5% sodium deoxycholate, 1 mM EDTA), and once in TE (10 mM Tris, 1 mM EDTA [pH 8.0]). After the last wash, 110 µl of elution buffer (50 mM Tris/HCl [pH 8.0], 10 mM EDTA, 1% SDS) was added to each sample, and the beads were incubated at 65°C for 10 min with periodic agitation. The beads were spun for 30 s at 10,000*g* at room temperature, and 100 µl of the supernatant was stored. A second elution was carried out with 150 µl elution buffer 2 (TE, 0.67% SDS), and eluates from the two elution steps were pooled (250 µl final volume). Both the ChIP and input samples were incubated overnight at 65°C, and cooled at room temperature. For cleaning the IPed DNA, 250 µl proteinase K solution (TE, 20 µg/ml glycogen, 400 µg/ml Proteinase K) was added to each sample, and samples were incubated at 37°C for 2 h. 55 µl 4 M LiCl was added to each, and the samples were extracted once with 450 µl phenol/chloroform/isoamyl alcohol solution (25∶24∶1). 1 ml ice cold 100% ethanol was added and the DNA was precipitated overnight at −20°C. The DNA was pelleted by centrifugation at 12,000*g* for 30 min at 4°C, washed once with ice cold 70% ethanol, and the pellets air dried. IP samples were resuspended in 25 µl TE, and input samples were resuspended in 100 µl TE+100 µg/ml RNaseA and incubated 1 h at 37°C. ChIP-enriched DNA was amplified, fluorescently labeled, hybridized, and washed as described in detail in Dataset S7. Labeled DNA for each channel was combined and hybridized to arrays in Agilent hybridization chambers for 40 h at 65°C, according to the manufacturer's instructions (Agilent Technologies). Arrays were scanned using Genepix 4000A Axon Instrument scanner. Analysis and identification of the binding events in the ChIP–chip data were determined as previously described [49] using Agilent Chip Analytics software v1.2 (Agilent Technologies). These binding events were displayed and analyzed using ChipView v0.954 (http://johnsonlab.ucsf.edu/). 250 bp centered on the midpoint of the peaks in the promoter regions bound by Zap1 were submitted to MEME v3.5.7 (http://meme.nbcr.net) for motif analysis [51] using the following parameters: minw = 7, maxw = 25, nmotifs = 10, maxsize = 50,000, mod = zoops. We also analyzed bound regulatory regions with the RSAT server, http://rsat.scmbb.ulb.ac.be/rsat/, using 1,500 bp of 5′ region sequence and a search for 8 bp motifs [52].

## Supporting Information

Dataset S1 ***C. albicans*** **strains used in this study.** This file gives the genotypes and sources for all *C. albicans* strains.
(0.06 MB DOC)

Dataset S2 **Oligonucleotide sequences**. This file gives the specific nucleotide sequence for each oligonucleotide.
(0.04 MB XLS)

Dataset S3 **pADH34 sequence.** This file gives the nucleotide sequence of vector pADH34, which was used for epitope tagging.
(0.01 MB TXT)

Dataset S4 **Microarray data**. This file gives complete microarray results for the comparison of the *zap1*Δ*/zap1*Δ mutant and *zap1*Δ*/zap1*Δ+*pZAP1* complemented strain (worksheet 1), a separate list of significantly regulated genes from this dataset (worksheet 2), and a comparison of Zap1-responsive genes in *C. albicans* and in *S. cerevisiae*, aligned as orthologs or best hits. Expression data for *S. cerevisiae* are from Lyons et al. [18].
(3.44 MB XLS)

Dataset S5 **Verification of Zap1-responsive gene expression.** This file provides data that support microarray results to indicate that Zap1-responsive genes are expressed at altered levels in the *zap1*Δ*/zap1*Δ strain and that the *TDH3* promoter fusion strains do indeed overexpress the relevant gene.
(18.31 MB DOC)

Dataset S6 **ChIP mapping of genomic Zap1 binding sites.** This file gives comprehensive mapping information for genomic Zap1 binding sites.
(2.98 MB XLS)

Dataset S7 **Detailed protocol for ChIP.** This file describes the protocol for ChIP used in this report.
(0.04 MB DOC)

## Abbreviations

ChIP: chromatin immunoprecipitation
CSLM: confocal scanning laser microscopy
