# Supplementary material for: Biofilm Matrix Regulation by Candida albicans Zap1
Source: PLoS Biol. 2009 Jun 16;7(6):e1000133. doi: 10.1371/journal.pbio.1000133 (PMC2688839; doi:10.1371/journal.pbio.1000133)
Supplement: Dataset S1 — C. albicans strains used in this study. This file gives the genotypes and sources for all C. albicans strains. (0.06 MB DOC) [file pbio.1000133.s001.doc]

| *C. albicans* strains used in this study | | |
| --- | --- | --- |
| **Strain** | **Genotype** | **Reference** |
| BWP17 | ura3∆::λimm434 arg4::hisG his1::hisGura3∆::λimm434 arg4::hisG his1::hisG | Wilson et al. 1999 |
| CJN1091 | ura3∆::λimm434 arg4::hisG his1::hisG zap1::ARG4ura3∆::λimm434 arg4::hisG his1::hisG zap1::URA3 | This Study |
| CJN1193 | ura3∆::λimm434 arg4::hisG his1::hisG::pHIS1-ZAP1 zap1::ARG4ura3∆::λimm434 arg4::hisG his1::hisG zap1::URA3 | This Study |
| CJN1201 | ura3∆::λimm434 arg4::hisG his1::hisG::pHIS1 zap1::ARG4ura3∆::λimm434 arg4::hisG his1::hisG zap1::URA3 | This Study |
| CJN1623 | ura3∆::λimm434 arg4::hisG his1::hisG::pHIS1 zap1::ARG4 PRA1::pAgTEF1-NAT1-AgTEF1UTR-TDH3-PRA1ura3∆::λimm434 arg4::hisG his1::hisG zap1::URA3 PRA1 | This Study |
| CJN1631 | ura3∆::λimm434 arg4::hisG his1::hisG::pHIS1 zap1::ARG4 IFD6::pAgTEF1-NAT1-AgTEF1UTR-TDH3-IFD6ura3∆::λimm434 arg4::hisG his1::hisG zap1::URA3 IFD6 | This Study |
| CJN1633 | ura3∆::λimm434 ARG4:URA3::arg4::hisG his1::hisG::pHIS1 ORF19.3449::pAgTEF1-NAT1-AgTEF1UTR-TDH3-ORF19.3449ura3∆::λimm434 arg4::hisG his1::hisG ORF19.3449 | This Study |
| CJN1638 | ura3∆::λimm434 ARG4:URA3::arg4::hisG his1::hisG::pHIS1 ORF19.4899::pAgTEF1-NAT1-AgTEF1UTR-TDH3-ORF19.4899ura3∆::λimm434 arg4::hisG his1::hisG ORF19.4899 | This Study |
| CJN1642 | ura3∆::λimm434 ARG4:URA3::arg4::hisG his1::hisG::pHIS1 ADH5::pAgTEF1-NAT1-AgTEF1UTR-TDH3-ADH5ura3∆::λimm434 arg4::hisG his1::hisG ADH5 | This Study |
| CJN1651 | ura3∆::λimm434 arg4::hisG his1::hisG::pHIS1 zap1::ARG4 ZRT1::pAgTEF1-NAT1-AgTEF1UTR-TDH3-ZRT1ura3∆::λimm434 arg4::hisG his1::hisG zap1::URA3 ZRT1 | This Study |
| CJN1655 | ura3∆::λimm434 arg4::hisG his1::hisG::pHIS1 zap1::ARG4 ZRT2::pAgTEF1-NAT1-AgTEF1UTR-TDH3-ZRT2ura3∆::λimm434 arg4::hisG his1::hisG zap1::URA3 ZRT2 | This Study |
| CJN1659 | ura3∆::λimm434 ARG4:URA3::arg4::hisG his1::hisG::pHIS1 YWP1:::pAgTEF1-NAT1-AgTEF1UTR-TDH3-YWP1ura3∆::λimm434 arg4::hisG his1::hisG YWP1 | This Study |
| CJN1663 | ura3∆::λimm434 ARG4:URA3::arg4::hisG his1::hisG::pHIS1 HXT5::pAgTEF1-NAT1-AgTEF1UTR-TDH3-HXT5ura3∆::λimm434 arg4::hisG his1::hisG HXT5 | This Study |
| CJN1667 | ura3∆::λimm434 ARG4:URA3::arg4::hisG his1::hisG::pHIS1 HGT2::pAgTEF1-NAT1-AgTEF1UTR-TDH3-HGT2ura3∆::λimm434 arg4::hisG his1::hisG HGT2 | This Study |
| CJN1675 | ura3∆::λimm434 ARG4:URA3::arg4::hisG his1::hisG::pHIS1 ORF19.999::pAgTEF1-NAT1-AgTEF1UTR-TDH3-ORF19.999ura3∆::λimm434 arg4::hisG his1::hisG ORF19.999 | This Study |
| CJN1680 | ura3∆::λimm434 arg4::hisG his1::hisG::pHIS1 zap1::ARG4 IFD4::pAgTEF1-NAT1-AgTEF1UTR-TDH3-IFD4ura3∆::λimm434 arg4::hisG his1::hisG zap1::URA3 IFD4 | This Study |
| CJN1684 and CJN1685 | ura3∆::λimm434 ARG4:URA3::arg4::hisG his1::hisG::pHIS1 ZAP1-Myc-FRT-SAT1-FRTura3∆::λimm434 arg4::hisG his1::hisG ZAP1 | This Study |
| CJN1688 and CJN1694 | ura3∆::λimm434 ARG4:URA3::arg4::hisG his1::hisG::pHIS1 ZAP1-Myc-FRTura3∆::λimm434 arg4::hisG his1::hisG ZAP1 | This Study |
| DAY185 | ura3∆::λimm434 ARG4:URA3::arg4::hisG his1::hisG::pHIS1ura3∆::λimm434 arg4::hisG his1::hisG | Davis et al. 2000 |

References:

**Davis, D., J. E. Edwards, Jr., A. P. Mitchell, and A. S. Ibrahim**. 2000. Candida albicans RIM101 pH response pathway is required for host-pathogen interactions. Infect Immun **68**:5953-5959.

**Wilson, R. B., D. Davis, and A. P. Mitchell**. 1999. Rapid hypothesis testing with Candida albicans through gene disruption with short homology regions. J Bacteriol **181**:1868-1874.
