# Supplementary material for: Biofilm Matrix Regulation by Candida albicans Zap1
Source: PLoS Biol. 2009 Jun 16;7(6):e1000133. doi: 10.1371/journal.pbio.1000133 (PMC2688839; doi:10.1371/journal.pbio.1000133)
Supplement: Dataset S5 — Verification of Zap1-responsive gene expression. This file provides data that support microarray results to indicate that Zap1-responsive genes are expressed at altered levels in the zap1Δ/zap1Δ strain and that the TDH3 promoter fusion strains do indeed overexpress the relevant gene. (18.31 MB DOC) [file pbio.1000133.s005.doc]

Supplemental file 5. Verification of Zap1-responsive gene expression

Quantitative real time RT-PCR measuring expression levels of *ZRT2, ZRT1, PRA1, IFD4, IFD6, ZAP1, ORF19.4899, ORF19.999,* and *ADH5* in the *zap1* mutant and reference strain DAY185 under biofilm conditions. Normalized gene expression values were calculated using the ΔΔCt method using *TDH3* as a reference gene. Results are the means of three determinations. For ease of interpretation, the reference strain expression level values were set to 1.0 for each gene set, and the normalized expression of each gene relative to the *TDH3* expression is shown.

Quantitative real time RT-PCR measuring expression levels of the relevant target genes in the *zap1* mutant, reference strain DAY185, and target gene overexpression (TGO) strains under biofilm conditions. Normalized gene expression values were calculated using the ΔΔCt method using *TDH3* as a reference gene. Results are the means of three determinations. For ease of interpretation, the reference strain expression level values were set to 1.0 for each gene set, and the normalized expression of each gene relative to the *TDH3* expression is shown.

Quantitative real time RT-PCR measuring expression levels of the relevant target genes in the *zap1* mutant, reference strain DAY185, and target gene overexpression (TGO) strains under biofilm conditions. Normalized gene expression values were calculated using the ΔΔCt method using *TDH3* as a reference gene. Results are the means of three determinations. For ease of interpretation, the reference strain expression level values were set to 1.0 for each gene set, and the normalized expression of each gene relative to the *TDH3* expression is shown.

Role of Zap1 and Zrt1/2 in growth on limiting zinc. Strains of genotypes indicated were grown at 30° on Synthetic Complete medium lacking ZnSO_4_ (upper panel) or Synthetic Complete medium (lower panel) for 2 days at 30°C. The specific strains used were DAY185 (*ZAP1/ZAP1*), CJN1201 (*zap1Δ/zap1Δ*), CJN1193 (*zap1Δ/zap1Δ*+*pZAP1*), CJN1651 (*zap1Δ/zap1Δ TDH3-ZRT1*), and CJN1655 (*zap1Δ/zap1Δ TDH3-ZRT2*).
