# Supplementary material for: Biofilm Matrix Regulation by Candida albicans Zap1
Source: PLoS Biol. 2009 Jun 16;7(6):e1000133. doi: 10.1371/journal.pbio.1000133 (PMC2688839; doi:10.1371/journal.pbio.1000133)
Supplement: Dataset S7 — Detailed protocol for ChIP. This file describes the protocol for ChIP used in this report. (0.04 MB DOC) [file pbio.1000133.s007.doc]

**Strand Displacement Amplification, Dye-coupling and Hybridization of Chromatin IP Samples**

**The following protocol uses high concentration exo^-^ Klenow (New England Biolabs #M0212M) and random DNA nonamers to perform strand-displacement amplification of the input and IP DNA samples from chromatin IP experiments. Prior to amplification, input and IP DNA concentrations are normalized by dilution of the input DNA for each corresponding IP based on qPCR values for a non-enriched locus, such as the ADE2 ORF (primers AHO294 and AHO295, Supplemental file 2). Input and IP samples are amplified separately, in parallel, and should yield similar amounts of product following each round of amplification. Typically, three rounds of amplification are required prior to dye-coupling and hybridization of the ChIP samples. If the IP DNA concentration is sufficient, the “round B” amplification step can be omitted. Since any DNA will be amplified by this approach, all amplification steps are performed with clean gloves, filter tips, autoclaved ddH_2_O and dedicated reagents which are free of any contaminating DNA.**

**Rnd. A (Primary amplification):**

**1) Mix: 12µl of IP sample or Diluted input (diluted in TE)**

**Note: equalize the input and IP samples based on qPCR values for a non-enriched locus**

**12µl H_2_O**

**20µl 2.5X SDA Buffer**

**2) Incubate 95^o^C, 5min then IMMEDIATELY transfer to WET Ice for 5min**

**3) Add 5µl dNTP mix (1.25mM each nucleotide)**

**4) Add 1µl 50U/µl exo^-^ Klenow (NEB)**

**5) Incubate 2hrs at 37^o^C w/ heated lid (in PCR machine)**

**Note: if needed, let sit up to ~2hrs at 10^o^C in PCR block or add 5µl 0.5M EDTA and store @ -20^o^C**

**6) Purify product with Zymo^25^ columns (Zymo Research): Add at least 3 volumes of binding buffer, bind, wash 1X with 200µl binding buffer, 2X with 200µl wash buffer, spin 1min at 10,000xG to dry and elute with 30µl H_2_O into a fresh tube**

**7) Check 1.5µl on a Nanodrop spectrophotometer (Thermo Scientific). If ≥400ng total, skip to Rnd. C. Otherwise continue with Rnd. B**

**Rnd. B (Secondary amplification):**

**1) Mix: 24µl Rnd. A DNA**

**20µl 2.5X SDA Buffer**

**2) Repeat steps 2-7 of Rnd. A, but elute with 50µl H_2_O**

**Rnd. C (aminoallyl-dUTP incorporation and final amplification):**

**Preferred approach:**

**100µl reactions with 2µg total Rnd. B DNA for each sample. Yields only ~2.5-3 –fold amplification, but dye coupling is still very efficient.**

1. **Mix: 1-2µg of Rnd. B DNA + H_2_O to 48µl total**

**40µl 2.5X SDA**

**2) Incubate 95^o^C, 5min then IMMEDIATELY transfer to WET Ice for 5min**

**3) Add 10µl 1.25mM aminoallyl-dNTP mix (1/10 dilution of stock sln)**

**4) Add: 2µl 50U/µl exo^-^ Klenow**

**5) Incubate 2hrs at 37^o^C w/ heated lid (in PCR machine)**

**Note: if needed, let sit up to ~2hrs at 10^o^C in PCR block or add 5µl 0.5M EDTA and store @ -20^o^C**

**6) Purify product with Zymo^25^ columns: Add at least 3 volumes of binding buffer, bind, wash 1X with 200µl binding buffer, 2X with 200µl wash buffer, spin 1min at 10,000xG to dry and elute in 50µl H_2_O**

**7) Check 1.5µl on Nanodrop… should yield ~5µg of total DNA per reaction**

**Alternate Rnd. C approach:**

**If Rnd. B yields less than 1µg total DNA, Set up 2X 100µl Rnd. C reactions for each sample, using 200-400ng of Rnd. B DNA per tube. Perform amplification and cleanup as described for the preferred approach, but pool the two independent reactions prior to Zymo^25^ column purification.**

**Dye Coupling:**

**1) Speed-vac amplified input and IP reactions from Rnd. C to ≤ 9µl total, or until dry**

**2) Re-suspend or QS to 9µl with H_2_O and add 1µl of fresh 1M NaBicarbonate, pH 9.0**

**Prepare NaBicarbonate on the day of labeling and pH with a pH meter, not strips**

**3) Immediately add 1.25µl Cy3 (input sample) or Cy5 (IP sample)**

**We use Amersham Mono-reactive dye packs (Cat.#PA23001 and PA25001). Each tube has enough die for up to 8 labeling reactions. Re-suspend the dye in 10µl DMSO and use 1.25µl of dye per labeling reaction. If fewer than 8 reactions will be performed, either decrease the volume of DMSO to use the entire tube, or aliquot and speed-vac the unused dye. Store any unused dye under desiccation at 4^o^C, protected from light.**

**4) Incubate labeling reactions for 1hr at 25^o^C in darkness**

**5) Add 800µl of Zymo DNA binding buffer to each sample and load onto a Zymo^25^ column**

**6) Wash 1X with 200µl DNA binding buffer, 2X with 200µl Wash solution, spin 1min at 10,000xG to dry, then elute with 50µl H_2_0 and check 1.5µl on a Nanodrop spectrophotometer using the “microarray” setting to quantitate the total yield and dye-coupling efficiency.**

**7) Proceed to the hybridization step, following Agilent’s protocol. Equalize the input and IP samples to 5µg each for a 1X244K array.**

**Note: Less DNA can be used…as little as 1µg each of input and IP samples have been successfully hybridized and scanned with no significant decrease in data quality**

**Hybridization Protocol (from Agilent oligo aCGH/Chip-on-Chip hybridization kit):**

**1) Mix 5µg each (input and IP) sample and bring volume to 150µl with H_2_0**

**2) Add 50µl of 1mg/ml Human Cot-1 DNA (Invitrogen)**

**3) Add 50µl of 10X Agilent Blocking Agent**

**4) Add 250µl of Agilent Hybridization Buffer**

**5) Mix thoroughly then quick-spin to collect**

**6) Incubate 3 minutes at 95^o^C then transfer immediately to 37^o^C for 30min**

**7) Spin 1 minute at full speed in microcentrifuge then carefully remove 490µl , load onto gasket slide, cover with array slide and assemble hybridization chamber**

**8) Hybridize for ~40hrs at 65^o^C**

**9) Disassemble the array and wash using Agilent wash buffers**

**a) 5min with mixing in Agilent Oligo aCGH/ChIP-on-Chip Wash Buffer 1 at 25^o^C**

**b) 5min with mixing in Agilent Oligo aCGH/ChIP-on-Chip Wash Buffer 2 at 32^o^C**

**c) 1min with mixing in Acetonitrile at 25^o^C**

**d) 30sec with agitation in Agilent drying and stabilization solution**

**Note: To ensure even drying, slowly remove the slide holder such that ~10 seconds elapse prior to complete removal from the solution.**

**Solutions:**

**2.5X SDA mix: (best if made fresh, but can be kept at -20^o^C for up to 1 month)**

**125mM Tris-HCl pH 7.0**

**12.5mM MgCl_2_**

**25mM BME**

**750µg/ml random DNA nonamers (dN9)**

**10X Aminoallyl-dNTP stock solution:**

**12.5mM dATP**

**12.5mM dCTP**

**12.5mM dGTP**

**5mM dTTP**

**7.5mM aa-dUTP**
